# Social Housing of Previously Single-Caged Adult Male Cynomolgus Macaques (*Macaca fascicularis*)

**DOI:** 10.3390/vetsci11110538

**Published:** 2024-11-01

**Authors:** Fitriya N. Dewi, Permanawati, Suryo Saputro, Adinda D. Azhar, Wahyu Putriyani, Jeffrey D. Wyatt, Irma H. Suparto, Huda S. Darusman

**Affiliations:** 1 School of Veterinary Medicine and Biomedical Sciences, IPB University, Bogor 16680, Indonesia; fitriyanur@apps.ipb.ac.id; 2 Primate Research Center, IPB University, Bogor 16151, Indonesia; permana1012@gmail.com (P.); ssaputro83@gmail.com (S.S.); adindaazhar@gmail.com (A.D.A.); wahyu.putriyani91@gmail.com (W.P.); irmasu@apps.ipb.ac.id (I.H.S.); 3 Department of Comparative Medicine, University of Rochester, Rochester, NY 14642, USA; jeff_wyatt@urmc.rochester.edu; 4 Department of Chemistry, Faculty of Mathematics and Natural Sciences, IPB University, Bogor 16680, Indonesia

**Keywords:** behavior, nonhuman primates, pairing, refinement, temperament, trio housing, welfare

## Abstract

Cynomolgus macaques (*Macaca fascicularis; Mf*) serve an essential role in the advancement of biomedical research. Aged macaques, in particular, are highly valued as animal models for the study of geriatric diseases. While social housing has become the default for nonhuman primates (NHPs), socially housing sexually mature males poses a unique challenge. Moreover, socially housing aged males that have been previously kept in single housing may pose even greater challenge and risk due to a higher likelihood of aggression. Temperament assessment plays an integral part in determining the success of social housing arrangements of NHPs. In this paper, we report our work at the Primate Research Center of IPB University in integrating behavior observation and veterinary management to socially house adult, male *Mf.* We describe our experience in successfully housing young-adult, male *Mf* (*n =* 8–10, aged 6 years) for over 2.5 years, and the multiple efforts to socially house a small group of previously singly housed aged, male *Mf* (*n* = 6, aged >15 years). The temperament of each *Mf* was considered in the planning and implementation of social housing attempts. While a simplified behavioral observation was performed for the young adults, a more rigorous PAIR-T assessment was performed on the aged *Mf*. A group formation was initially attempted, aiming to achieve a small group of six aged, male *Mf*. While this group at first showed promising outcomes, significant incidents of aggression required regrouping as pairs or triads wherein the subject combinations were determined based on temperament and dominance. A total of three attempts were made to house these aged *Mf* in a small group throughout the course of 17 months, and the results showed that at our facility, aged, male *Mf* are best housed as triads or pairs, with selections based on their temperament and dominance compatibility. We concluded that behavioral assessments and veterinary management are pivotal in supporting the social housing efforts of adult, male *Mf* in order to optimize their well-being.

## Simple Summary

Nonhuman primates (NHPs), such as cynomolgus macaques, have contributed significantly to biomedical research. Socially housing mature male NHPs presents challenges, especially if they were previously singly housed. Temperament assessment is crucial for successful group-housing of macaques. At IPB University’s Primate Research Center, efforts were made to house a small group of male cynomolgus macaques by integration of temperament assessments, behavioral observation and continuous veterinary oversight. While social housing of young-adult males was achieved for over 2.5 years with relatively few incidents, aged macaques which were initially housed in a group of six were maintained best as pairs or trios based on their temperament and dominance compatibility. This study showed that behavioral observation and veterinary management are critical in ensuring the success of social housing efforts.

## 1. Introduction

Nonhuman primates (NHPs) such as macaques have greatly contributed to the field of biomedical research. NHPs’ sentience and cognitive capacity warrant efforts to ensure and promote their psychological well-being in captive settings. Bioethical approaches have evolved significantly over the years, driven by a combination of regulatory changes, advancements in research and increased understanding of NHP behavior and welfare. One of the fundamental changes has been the recognition of the importance of social housing and environment for the enrichment of species-specific behaviors. Social housing is one of the most basic necessities for the improvement of the psychological well-being of NHPs [1,2]. Cynomolgus macaques (*Macaca fascicularis; Mf)* are among the most valuable NHP species in research and testing [3,4]. Members of this macaque species invest significant amounts of time in social behavior such as allogrooming, and require companionship to provide social buffering and reduce stress [5]. Singly housed *Mf* readily exhibit abnormal behavior patterns, resulting in biased physiological parameters in research studies [6]. Contrastingly, social housing has a positive impact on the welfare of captive macaques by reducing abnormal behaviors and improving interactions, physical activity, play and exploration [7,8]. Social housing provides opportunities for primates to engage in affiliative behaviors, which are essential for their well-being and species-typical behavioral repertoire [9].

Researchers strive to improve the welfare and ethical standards of NHP care and use. The *Guide for the Care and Use of Laboratory Animals* and AAALAC International indicate that social animals should be housed in stable pairs or groups of compatible individuals unless other arrangements are justified by experimental or veterinary-related concerns [10,11]. The U.K. regulations and European Union directive also emphasize the necessity of the social housing of social species, mandating that that NHPs be housed in groups unless there are specific reasons for exemptions [12,13]. Therefore, behavioral management programs often prioritize increasing the proportion of socially housed primates to enhance their welfare [1]. Although single housing of NHPs should be avoided whenever possible, social housing is not without challenge. Aggression, trauma and the need to mitigate dominance hierarchies can arise [14]. While transitioning juvenile and young-adult macaques from single-housing to group-housing may be less risky, the challenge is magnified with adult males [15]. The risks of aggression, incompatibility and injury pose real concerns when facilities face the limitations inherent in acquiring aged macaques for geriatric studies such as those involving neurodegenerative diseases and other disorders affecting millions of people worldwide [16]. Pairing adult male macaques can be challenging [17], and moreover, successful pairing of older macaques greater than 17 years old has been infrequent [6]. At the same time, there is trend of increasing amounts of research on aging, which reflects the growing recognition of the health complexities associated with the aging human population [18]. This trend suggests the important role of aged macaques as models to support this research field, and appropriate housing and management strategies are imperative for their well-being.

Behavior management strategies, such as environmental enrichment, temperament assessment and positive reinforcement training play a significant role in reducing stress and facilitating veterinary care for nonhuman primates [19,20]. Behavior assessment is a crucial component for the social housing of macaques in captive settings. By evaluating the behavior of macaques, researchers and veterinarians can gain insights into the macaques’ social interactions, preferences and compatibility which are essential for successful social housing arrangements. Understanding the behavioral profiles of individual macaques can aid in assessing their temperament, which helps in determining suitable social partners, introducing them effectively and monitoring their compatibility over time. Behavioral management interventions, guided by behavior assessment, can help alleviate potential issues between social partners, ensuring a harmonious social environment for macaques and improving the health care of the animals [6]. It is therefore important to integrate a behavior management component into the veterinary care program. Here, we report our experience of incorporating a temperament assessment approach in socially housing a group of previously singly housed 16- to 18-year male *Mf* in a group of six, as pairs and triads, along with a similar effort involving ten 6- to 7-year-old *Mf*.

## 2. Materials and Methods

### 2.1. Ethical Statement

All procedures were approved by the IACUC of the Primate Research Center at Bogor Agricultural University (IPB University), with protocol approval numbers IPB PRC-19-B001, IPB PRC-19-B005 and IPB PRC-19-B008. The procedures were performed at the Research Animal Facility—Lodaya (RAF-L) of the Primate Research Center at IPB University (PRC-IPB), an AAALAC International-accredited institution in Bogor, Indonesia. Housing and procedures complied with the U.S. National Research Council (NRC) *Guide for the Care and Use of Laboratory Animals* and Indonesian regulations and guidelines [21].

### 2.2. Animals

As to the young adult intact male *Mf*, ten 6- to 7-year-old, cynomolgus macaques (*Macaca fascicularis; Mf*) of Indonesian origin (Subjects A to J) were acquired from the harem breeding site of PRC-IPB, where they were group-housed upon weaning. The harem breeding colonies for *Mf* were composed of a 1:10–15 ratio of male and female breeders, housed in gang cages or larger corrals. After being weaned, these males were housed in multi-male, multi-female groups until reaching maturity or breeding age, and then moved to multi-male groups until further enrolled in a study or assigned to a breeding colony. The small groups typically consist of no more than 15 subjects, all of similar age and size, as adjusted based on housing size. The criteria for enrollment in this study were their SPF status (TB-free and retrovirus-free) and clinical health status based on routine physical examination. These macaques were then singly housed for 9 months while in a study at the RAF-L of PRC-IPB before enrollment in the social housing effort. Body weights were measured before introducing animals and then periodically re-measured during regular clinical examinations for almost three years.

As to the aged intact male *Mf,* six *Macaca fascicularis (Mf*) of Indonesian origin, each estimated to be 16 to 18 years old based on dentition, were adopted by the PRC-IPB from a local CRO in Indonesia, where they were singly housed for more than 5 years. The local CRO specialized in NHP studies, although the programs did not exclusively involve NHPs. The animals were SPF (TB-free and retrovirus-free), and identified as spontaneously pre-diabetic (*n* = 3) or non-diabetic (*n* = 3) based on their blood glucose values. Upon arrival, the animals were singly housed during a 30-day quarantine. Body weights were measured before introducing animals and then periodically re-measured during regular clinical examinations for over 17 months.

### 2.3. Housing and Husbandry

**Figure 1:**
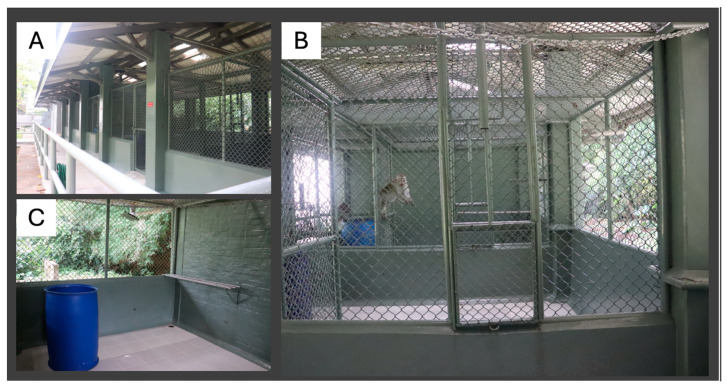
Semi-outdoor cages used for social housing. Interconnected pens were used in the study (**A**). The pens were equipped with sliding-door dividers and separated by diamond-shaped bars, which allows tactile contact between animals in adjacent pens (**B**). Each unit had multiple perches, barrels providing visual barriers and stainless-steel chains as structural enrichment (**C**).

All subjects were fed a standard commercial NHP diet (Charoen Pokphand, Bangkok, Thailand) twice daily, with ad libitum access to water, supplemented daily with a variety of fruits. All animals experienced the ambient humidity, temperature and photoperiod. Group-housing attempts were made in semi-outdoor, sheltered, epoxy-coated and galvanized gang cages with tiled floors (Figure 1). The housing consisted of three interconnected pens that could be separated using sliding-door dividers, each unit being 2.25 m high with a 10.41 m^2^ floor area. The pens were separated by diamond-shaped bars providing 15 cm^2^ of space between the bars for tactile contact among animals in adjacent pens. Each unit had multiple perches, in addition to barrels, which provided visual barriers, and stainless-steel chains for climbing and swinging. Two adjacent pens were used for the social housing attempts (i.e., as a small group or trios) while one pen was dedicated to housing individual cages for emergency use. However, all three pens were occupied when aged animals were housed as pairs. The individual cages available for emergency single-housing were 87 cm high with a 4.14 m^2^ floor area, and used only for clinical cases. The singly housed animals under clinical care maintained visual and auditory interaction with other group-housed macaques in the adjacent pen.

### 2.4. Temperament Assessment and Criteria for Identifying Social Housing Candidates

The behavioral temperament of the young cohort of *Mf* was determined by evaluating their reactions during cage-side observation and when hand-fed, and then subsequently characterized as fearful, non-aggressive or aggressive. This simplified method was chosen based on previous successful experiences with the grouping of young cohorts.

In consideration of the higher risk involved in grouping a cohort of aged males with a long history of individual housing, the temperament assessment of individuals in the older cohort used a more detailed PAIR-T methodology, which is a modified version of the human-intruder test [22]. Two personnel independently conducted PAIR-T assessments over 10 days. The assessor posing as a human intruder observed the macaque’s reaction for one minute while facing away from the animal followed by one minute staring at the animal. Key behaviors categorized each macaque as having one of five different temperaments: aggressive, neutral, affiliative, anxious and fearful. Based on the rates of the observed behaviors, the temperament of each animal was characterized. Upon social grouping, temperament was re-assessed by evaluating reactions during cage-side observation and when the animals were hand-fed.

The results of individual temperament assessments were used to determine the order of social introduction of the *Mf*s in the formation of the social group. Importantly, the temperament categories were used as criteria when aged *Mf* were later paired or kept in triads. Selection of potential trio or pair partnerships was based on temperament categories determined by PAIR-T pre-assessments, wherein an anxious monkey was paired or grouped with neutral or affiliative ones, but not with the aggressive. Planning of trio- or pair-housing also took into consideration the social dominance that was observed or monitored upon each social housing effort.

### 2.5. Social Introductions

Prior to social introduction, the animals were housed in individual cages located in the same room, with cages facing each other to ensure visual and auditory contact among them. These cage racks were then moved into an area adjacent to the pens to be used for social housing to allow for adaptation to the environment. This adaptation was performed for at least 10 days.

For the young-adult, male *Mf*, the objective was to form a social group comprising all of the subjects. The social introduction of members of the young cohort from single cages to the group-housed setting was performed in rapid succession by opening one cage door at a time. The introduction was performed according to the temperament category in the following order: fearful and smallest *Mf*, non-aggressive *Mf* (small to large) and aggressive *Mf* (small to large). This method allowed full contact among animals immediately upon their release to the pen. Upon release, the racks of individual cages were removed to allow the group of ten animals to freely occupy both interconnected pens.

For the aged, male *Mf*, the initial objective was to form a small social group comprising all six subjects. In cases in which clinical incidents occurred in ways that indicated social incompatibility, the individual temperament data were then used to determine subsequent trio or pairing strategies. The process of the social introduction of the aged cohort was also accomplished by opening cage doors in rapid succession, allowing immediate full contact. Similar to the process used for the younger adult males, the social introduction process started by placing racks of individual cages inside one of the interconnected pens. Animals were released from individual cages, with the order being determined according to their temperament category, in the following order: anxious, neutral, affiliative, and aggressive.

Upon completion of the social introduction process, at least two veterinary care and husbandry personnel remained present at cage-side for a minimum of 15 min to anticipate the need for immediate intervention. In the absence of incidents or aggression, the personnel gradually moved away from the cage-side position to a more distant site to perform observations within the subsequent 30 min. Given the semi-outdoor nature of the cages in the animal housing area, distant observations were able to be performed intermittently by at least one personnel for the next 2 h (i.e., animals observed every 30–45 min). Following the absence of signs of aggression during this observation period, personnel were then allowed to leave the housing area.

### 2.6. Monitoring

In addition to the performance of regular daily health and husbandry rounds, macaque behavior and their interactions with other individuals were observed, for a minimum of 15 to 30 min duration, at least 3 times per day, between 8:00 a.m. and 4:00 p.m., following every grouping attempt. These ad libitum cage-side observations were performed for at least 3 days after each grouping, with particular attention given to affiliative or aggressive behaviors. The temperament and dominance of each subject were determined by these observations, and during attempts to hand-feed. The dominance levels between animals were determined based on affiliative interactions observed between the subjects, which were categorized as dominant or subordinate, and the subordinates were defined further as higher and lower in rank. These observations were performed mainly to provide additional factors that could be considered in the planning of trio- or pair-housing as an alternative when social introduction into small groups indicated social incompatibility.

Importantly, all injuries and wounding-related events were documented and categorized as minor, moderate or severe based on a veterinary judgment of the size, number and nature of the lesions (e.g., redness, bruise, swelling, or laceration with or without active bleeding); condition of the animal (e.g., remained bright/alert/responsive; impaired in movement and/or use of limbs or other body parts; or exhibited signs of pain, appeared lethargic and demonstrated inappetence); and requirement for clinical intervention (e.g., wound treatment; the use of drugs such as analgesics or anti-inflammatory medication; fluid therapy; the use of antibiotics; and suturing, as appropriate). Minor woundings were defined as those that did not require medical intervention. While both moderate and severe injuries warranted clinical intervention by the veterinary team, severe wounding was categorized as those injuries requiring more than five days of treatment, which may or may not involve isolating the injured animal(s) from the group. Isolation in such clinical cases, when performed, utilized emergency single-housing located inside a pen that was adjacent to the group pens.

### 2.7. Data Analysis

Body-weight data was analyzed using the one-factor repeated-measures model in JMP version 12 (SAS, Cary, NC, USA). All observational results are discussed descriptively and summarized in the tables below.

## 3. Results

### 3.1. Young-Adult Mf Cohort Compatibility

The profile of the young-adult *Mf* cohort is presented in Table 1. The social housing attempt started with the introduction of ten subjects into a group, though two animals were removed for a study after 6 months, resulting in a cohort of eight. Following a severe injury in one animal that happened after one year, the group was reduced to 7; this group lived together for several years.

Statistical analysis indicated a significant change in body weight over time (*p* < 0.0005). While some animals showed a marked and consistent increase in body weight over the 2.5 years, others experienced a more transient increase. Decreased weights were found intermittently for certain timepoints in a few animals; these were related to events involving significant wounding or injury. In addition to immediate clinical intervention during aggressive incidents, support such as fluid therapy and provision of vitamins was also given during regular check-ups, based on the veterinarian’s judgment. Aside from the wounding-related events, the animals were, overall, in good clinical condition throughout the course of their socially housed periods.

Minor altercations were immediately observed upon group formation, but none required clinical intervention. A summary of clinical incidents that occurred during the process of grouping the young cohort of male *Mf* and throughout their social housing period is presented in Table 2.

Within the first week of group formation, several minor wounding-related events occurred—mainly involving Subjects A and C, who were of fearful and aggressive temperament, respectively. Within the first month of being socially housed, aggression and wounding-related events were intermittently observed involving almost all *Mf* in the group. Subject A appeared to be the main object of aggression by others. Although some wounding-related events required clinical intervention, none warranted removal of the *Mf* from the colony during this time. Reduced aggression was observed by the second month and through the fourth month of social housing. Subjects G and I, who were identified as aggressive and non-aggressive, respectively, appeared to be more dominant in the group across this period of observation.

In month 6, Subjects B and F were removed from the group for another research project. As the number of animals was reduced to eight, it was decided that only one pen was needed to house the colony. Limiting housing to one pen resulted in minor aggression, mainly involving Subjects G and I as the dominant individuals, and C as the regular target. At one point the injury in Subject I was significant, requiring clinical intervention. The dividing door was then opened to allow access to two pens, which resulted in less aggression and increased group stability.

After one year of observation, the colony of eight *Mf* remained as a stable group, with a few minor aggression- and wounding-related events. However, subject H, an individual identified as aggressive, was permanently removed from the group at month 15 due to severe injury. The remaining seven *Mf* had a relatively stable interaction wherein intermittent aggression still occurred, but only a few of these required clinical intervention. Approaching the third year of social housing, all seven animals were re-assigned to several different protocols, permanently splitting up the group.

### 3.2. Aged Mf Cohort Compatibility

The profile of the aged *Mf* cohort is summarized in Table 3. The process of social introduction for the aged cohort started with the anxious animal (Subject 6), who did not immediately enter the gang pen and was hiding around the racks until all animals were released. All six subjects initially occupied two interconnected gang cages.

While the group appeared stable upon the initial release and during the period of immediate intensive observations over three hours, intermittent altercations started to occur when personnel left the area. During the first week, a total of four subjects were involved in several wounding-related events, requiring Subject 4 (of neutral temperament) to be moved to individual housing to receive clinical treatments in the adjacent pen. In the second and third weeks following grouping, intermittent but mild aggression still occurred involving various animals—mainly found in Subjects 5 and 6, who were of affiliative and anxious temperament, respectively. At one point, Subject 1, an aggressive subject, had to be removed to receive clinical treatment in individual housing, leaving the colony as a small group of four subjects occupying two interconnected gang cages. After the clinical treatments of Subjects 1 and 4 were completed, planning for regrouping was formulated.

The progress of the colony formation, clinical incidents and subsequent regrouping attempts are summarized in Table 4.

The regrouping attempt was performed by pairing. All animals were housed in pairs based on their initial PAIR-T assessment as well as their observed post-grouping temperament. Level of dominance, which was determined based on affiliative interactions between the subjects upon grouping (Table 5), was also taken into consideration in the pairing attempts. Pairings of individuals were performed with the following combinations: aggressive–affiliative, neutral–anxious and neutral–neutral. Each of the three pairs occupied one pen but contact with neighboring pairs was still observed, given the nature of the caging. These pairs were relatively stable, demonstrating mostly affiliative behaviors, with a few aggressive interactions. Although these pairs were promising, four months subsequent to the first introductions, another attempt of regrouping, as triads, was made to reduce the number of pens dedicated to this project. The two triad groups continued to exist, with only minimal incidents, during the following month of observation.

In the fifth month since first introductions, a second attempt of group-housing all six animals was conducted by opening the dividing door between the two pens. Within a few days, however, minor aggression escalated into the wounding of a majority of the animals. Therefore, animals were returned to the successful triad grouping achieved beforehand. Although aggression still occurred upon regrouping into the triads, the interactions gradually improved. This stable trio-formation lasted for almost three months before a grouping of all six was attempted again.

The third and final attempt of group-housing was attempted at eight months following the first social introductions as a group of six. Aggression initially occurred, although it required no intervention. Although relatively stable interactions were observed for approximately one month, additional episodes of significant aggression warranted a return to triad housing. The triads, however, were formed with different group compositions: Subjects 2-4-6 and 1-3-5. The composition took into consideration the dominance or social rank that had become more evident over time. This composition of triads exhibited a minimum of incidents, mostly affiliative behaviors, with the exception of intermittent conflict between the neighboring groups through the pen dividers, with no significant wounding associated with this conflict. The stable triads lasted for more than six months. Approaching month 17 after first introductions, despite the stability of the colony, Subjects 2 and 3 started to show signs of a deteriorating condition related to progressive diabetic metabolic syndrome. After removal of these two individuals from their triad for clinical care, four animals remained as compatible pairs. The remaining pairs were 4-6 (neutral and anxious), and 1-5 (aggressive and affiliative).

Over the course of a 15-month observation, the aged *Mf* were found overall to be in good clinical condition and with normal appetites, except during periods of significant aggression that resulted in injury or wounding, which mainly happened during initial group formations and less so in triads. There were significant changes in body weight over time in these aged animals (*p* < 0.0001). Subjects 1, 5 and 6 showed mild, intermittent decreases in body weight, whereas Subjects 2, 3 and 4 showed gradual decreases that occurred regardless of wounding-related events. This might be due to the fact that the latter animals had pre-existing pre-diabetic profiles. Notably, Subjects 2 and 3 exhibited deteriorating conditions which became more evident by month 17, as supported by their high blood glucose values, despite a stable interaction with others in the colony, as indicated by the lack of aggression- and injury-related events since the formation of the last triads. This clinical condition was likely due to a progressive diabetic metabolic syndrome. These two *Mf* were removed from the group for intensive observation, clinical treatment and eventual euthanasia due to poor clinical prognosis.

## 4. Discussion

Although challenging, we found that social housing of previously singly caged adult male cynomolgus macaques (*Mf*) was feasible. This study showed that young-adult, male *Mf* were successfully group-housed as a small colony of seven, almost without significant aggression; this was achieved due to continuous behavioral and veterinary oversight. Importantly, our observation showed that the aged *Mf* males were best housed as trios or pairs. While the younger adults did not require rigorous pregrouping behavior assessments to achieve a stable group, planning a social housing of aged males required careful considerations of their temperament and dominance and provision of continuous oversight by the veterinary care team for each grouping attempt. Back-up housing strategies are important during group formation, in case of failures. To our knowledge, this report is the first to describe a social housing process for aged, male *Mf* (i.e., >15 years of age), and particularly those that have been previously singly housed for the majority of their lives. Grouping of aged males with various temperaments was attempted. Following aggressions that led to varying degrees of injury upon formation of the small colony, regroupings into pairs and trios were performed with various combinations, determined according to their temperaments and dominance, until a suitable pairing or grouping was achieved.

Social housing strategies of macaques have been increasingly reported in recent years, in synchrony with the overall effort to improve the welfare of NHPs used in research. A recent report on behavioral management programs for laboratory NHPs in the United States showed that 83% of the *Mf* population across 23 facilities were socially housed, whereas only 77% of *Mf* maintained indoors for research purposes were socially housed [1]. In addition to the guidance in the NRC’s *Guide for the Care and Use of Laboratory Animals*, which recommends avoiding the single housing of social species [10], the guidelines of the Association of Primate Veterinarians (APV) further elaborate on the important aspects and considerations involved in socially housing NHPs. The APV guidelines and several regulatory bodies in the U.S. and U.K. suggest that NHPs of a similar health status used in studies of infectious disease, vaccine efficacy and surgical implants, and those requiring tethering or use of jackets, should still be socially housed, as it does not compromise study validity [23,24]. Macaques are social beings, and therefore, social housing of these animals enhances their ability to cope effectively, exhibit natural behavior and have a balanced temperament with less chronic distress [25], which are all important in supporting the validity of studies. Macaques kept in single housing exhibit abundant abnormal behavior that varies from self-plucking manifesting as alopecia to stereotypy and self-injurious behavior [2,19]. Although the benefits of social housing and the negative impact of individual housing have been well established for macaques, the practice of socially housing adult male NHPs is known to be challenging. The reasons commonly given for not attempting social housing are characterized by the overestimation of the risks and underestimation of the benefits [25].

The main challenge in implementing social housing of NHPs is social incompatibility, which may be indicated by fighting or aggression among individuals that results in injury or distress [1]. Our results showed that several aggressive events occurred in association with social housing attempts wherein wounds of various severity were found, with some requiring separation of the animal(s) in order to receive clinical treatment prior to being regrouped. These incidents happened more prominently with the aged male macaques, and in the initial stage of grouping. Although this finding demonstrated that harmful conflicts, a typical justification for single housing, was in fact a valid concern, such initial aggression should not be viewed as an absolute discouragement. It is important to integrate behavior monitoring into the veterinary care program in every grouping attempt in order to promptly decide upon intervention strategies. Careful consideration should be used in the decisions to tolerate aggressions, to provide medical treatment while maintaining the colony, to remove individuals, or to further break up the group into smaller subgroups or pairs based on temperament combination and dominance considerations. Macaques are social animals that naturally live in multi-male, multi-female groups, and *Mf* in particular have a hierarchy system that determines the dynamics of the colony. It is known that aggression among adult males is common in the natural setting, especially when new males join a colony or when there is a replacement among the high-ranking males [26]. Aggression may readily occur when housing multiple adult males in captivity. It is important to evaluate early behavior during social introductions, as it may predict pairing success in male macaques [27]. The absence of significant aggression during the introduction period is often associated with stable pairing [6], but it is important to note that initial incompatibility and injuries are not always indicative of pairing failure [23].

Social housing attempts with different species and age groups of macaques have different levels of risk, and there is no single approach that will work for all species of all animals at an institution [22]. Understanding individual differences in temperament or personality among macaques is essential for identifying compatible pairs, determining introduction processes, and reducing conflicts in group settings. For example, adult female macaques of similar temperaments may be more compatible with each other and exhibit interactions which are less aggressive, while forming stable male pairings may require adult macaques of dissimilar temperaments [6,22]. Personality dimensions and their interplay within a social hierarchy may also predict variations in social relationships [28]. Therefore, temperament assessment is a crucial step to aid in the selection of suitable partners for social pairing and grouping, and this process requires expertise in assessing NHP behavior. While pre-planning is critical, the process needs to remain flexible, recognizing the need to improvise based on the states of the animals involved [22]. It is important to consider that factors such as weight disparity, behavior patterns and environment also contribute to the complexity of social compatibility. Furthermore, any attempt to socially house adult males requires a commitment to provide consistent monitoring by dedicated personnel, combining veterinary care and behavior management to allow for timely separation of animals and/or clinical intervention. Continuous monitoring of behavioral welfare measures can help gauge the success of social housing arrangements and identify areas for improvements that enhance the socialization experience for macaques [8].

Social housing processes that have been reported in recent years have mainly been pairing-related. The species involved were mostly rhesus macaques (*Macaca mulatta*), although pairings of other macaques such as *Mf*, stumptail (*Macaca arctoides)* and pigtail (*Macaca nemestrina)* have also been reported [15,29]. A recent work in rhesus macaques supported the idea that trio housing may be a promising approach for socially housing NHPs and posited that careful consideration in the introduction strategy is crucial [30]. *Mf*, as a species, is known to be more aggressive during social-housing attempts than are rhesus [15]. Here, we have demonstrated that aged, male *Mf* adapted relatively well with the trio housing approach when performed taking into consideration the results of temperament assessments. Although aggressive behavior was intermittently visible, we found that the aged colony was relatively stable as triads, suggesting the importance of temperament and hierarchy identification in pre-determining grouping and introduction strategy among the older male *Mf*. We found that, although the PAIR-T method was designed for pairing, it was useful in providing guidance for determining trio-housing strategies, such as to avoid the housing of aggressive subjects with anxious subjects, or to choose a grouping of an aggressive individual with neutral or affiliative subjects. It was also our goal to form a group which had a high likelihood of immediately achieving a clear or marked social hierarchy, as rank instability is a likely cause of aggression used to establish social dominance. Social housing of male macaques as a small group will naturally result in the establishment of social ranks, and aggression tends to occur when the hierarchy has not settled [31]. It has been reported that an early onset of submissive behaviors during introduction of pairing is associated with lower wounding [32]. One limitation of our study was the lack of behavior-data quantification associated with social housing attempts, such as data acquired with focal animal sampling or scan sampling. Although intensive observations were performed to identify social compatibility upon grouping, these were mainly performed from a veterinary care perspective, and no quantifiable data were collected in an effort to systematically aid future compatibility assessment.

The process of introducing multiple adult, male *Mf* into a social group setting in captivity must be carefully considered and planned. There has been a relatively low consistency in terms of the methodology of social housing efforts across different facilities, particularly with respect to the introduction process [1]. A number of facilities implemented a step-wise introduction process, involving gradual steps from visual contact to protected contact, before placing the animals in unimpeded free contact. Gradual steps typically aim to minimize injury risk, but are labor-intensive and may not fully predict aggression until animals are in full contact [1,6]. Various introduction processes have been elaborated in great detail for pairing, whereas less information can be found about the introduction methods used for group formation. For example, there were studies that reported group formation of male *Mf* of a wide age range (5–15 years) [31] or young adults (4–6 years) [33] using a step-wise social housing process to form small groups of four *Mf*. In contrast, there have been studies that used a rapid-grouping approach for the formation of small groups of three adult, male *Mf* (10–15 years of age) [34] or a small group of seven young-adult *Mf* (aged 4–5 years) [35]; these were similar to the approach we implemented. We selected a rapid-grouping approach based on logistical and practical considerations. Also, the labor-intensive process of periodic and step-wise introductions among 6 to 10 animals may not necessarily predict compatibility or serious injury when animals are placed together in full contact. Risk associated with our rapid-pairing process was mitigated by using temperament assessments to determine both the order of animal release from individual cages into a group of six as well as individuals likely to succeed as triads, and provision of consistent observations by multiple personnel prepared to intervene. Given the small sample-size reported herein, these results may be specific to our facility, and further research with larger sample sizes would be needed to understand the dynamics during the introduction of animals into trio housing in order to predict the likelihood of success for stable colony formation as well as to identify potential factors that may lower the risk of aggression.

NHPs are important for geriatric studies due to their physiological, genetic and behavioral similarities to humans. Importantly, the lifespan of macaques can reach up to 27 years in captivity, which allows for studies on age-related changes that parallel human aging processes [36]. These macaques, however, are typically acquired as aged animals, which poses significant risk/benefit propositions when considering social housing. While our experience using a simplified approach for grouping young-adult, male *Mf* showed a good outcome, we decided that a more strategic approach involving specific behavior assessment was warranted to carefully plan the social housing of aged, male *Mf*. Our social housing of seven sexually mature (young adult) macaques lasted for more than 2 years. Injuries occurred mainly during the initial stage of grouping, emphasizing the need for intensive veterinary care and behavior monitoring, especially during this period, until the colony stabilizes, as indicated by a state of less aggression. Although incidents that resulted in injury were still observed intermittently over time, they were mainly of minor to moderate degree of severity. The pattern of the animals’ body-weight gain was consistent with a previous report that detailed a delayed gain upon group-housing, with an association found between social rank and body-weight gain over time [35]. These young-adult, male *Mf*s had been exposed to social experiences throughout the majority of their lifetimes, which differed from the members of the aged, male *Mf* colony. The younger males had experienced single housing, but only for a relatively short period, and had otherwise acclimated to social housing throughout their lifetimes. The success of group-housing macaques is significantly influenced by their social experience, as it plays a vital role in facilitating integration into future social groups. Exposure to social interactions and group dynamics early on can enhance adaptability and social skills, contributing to successful group-housing arrangements for macaques [37]. The formation of small groups of the older or aged macaques, however, occurred with significant aggression. The aggression-related events were likely related to the fact that the establishment of hierarchies undergoes strengthening through adulthood, whereby social interactions to claim social ranks occur prominently with age and often involve aggression [38]. Moreover, the history of prolonged single housing associated with the aged, male *Mf* in our study contributed to the significant challenge of social integration and compatibility occasioned when transitioning to group settings, as such a history could result in macaques’ lacking the social skills and experience necessary to interact effectively with conspecifics [39]. Although age and the duration of previous single housing of individuals seemed to be important factors in the formation of a stable social group of male *Mf*, our study was not specifically designed to directly compare the process and outcomes between young-adult *Mf* and aged *Mf*, which limited us from analyzing these dynamics further. Here, we have reported the observations of social interactions following grouping attempts involving young-adult *Mf* and aged *Mf* based on our experience with these colonies, but more systematic research is needed to evaluate the roles of temperament, personality and social hierarchy as determinants of group formation in adult males of different age groups. The successful social grouping of *Mf* is likely found in a complex interplay of all these factors, and understanding these dynamics will be important in improving the grouping process.

Socially housing macaques with compatible companions is a complex task that requires a deep understanding of species-specific social behavior and experience in managing social dynamics [40]. Behavior observation plays a key role in evaluating the impacts of social interactions on the psychological well-being of macaques. It is important to perform continuous behavior assessment and management to support appropriate veterinary interventions that can enhance the well-being of macaques [41]. Monitoring behaviors related to temperament types, aggression, and social interactions allows veterinarians to plan a social housing strategy, evaluate its effects on macaques and implement more efforts to enhance their overall welfare. We demonstrated considerable success in forming pairs or triads of aged, male *Mf* with histories of being singly housed, as indicated by the observed social compatibility with less aggression. Importantly, we suggest that behavior assessment allows for safer pairing and trio housing of aged individuals. Although formation of a small social group of aged *Mf* may still serve as the ultimate goal, it would require further behavior observation and thorough temperament, personality and hierarchy assessments to allow for more careful planning and the development of a management strategy to achieve social compatibility. By evaluating behaviors, identifying temperaments, addressing compatibility issues, and promoting positive social interactions through behavioral management strategies, the social environment of the macaques may be enriched to benefit their psychological well-being and welfare.

## 5. Conclusions

While a stable small group of previously singly housed male *Mf* may reliably be formed during the period from its members’ young-adult years to their adulthood (aged 6–9 years) without rigorous behavioral assessments, our results suggest that pregrouping assessments of temperament and further evaluations of social dominance or hierarchy best position institutions for success when housing aged (>15 years old), male *Mf* as pairs or triads. Behavioral and veterinary management is important in the planning and monitoring of every social housing attempt involving adult, male *Mf* in order to achieve social compatibility and minimize clinical risks.

## Figures and Tables

**Table 1:** Temperament of each young cohort subject, based on cage-side observations and/or hand-feeding reactions, and body-weight profile.

Subject ID	Temperament	Pre-Social Housing Body Weight (kg)	Post-Social Housing Body Weight (kg)
A	Fearful	5.05	5.86
B	Non-aggressive	5.44	5.14 ^1^
C	Aggressive	5.74	7.46
D	Non-aggressive	8.61	8.24
E	Aggressive	5.03	5.12
F	Non-aggressive	5.78	5.27 ^1^
G	Aggressive	7.56	8.54
H	Aggressive	5.24	5.14 ^2^
I	Non-Aggressive	6.47	5.39
J	Non-Aggressive	5.87	6.28

^1^ Subject was removed from the colony for another project at month 6. ^2^ Subject was removed from the colony due to clinical justifications at month 15.

**Table 2:** Clinical incidents documented in the young cohort of males upon grouping.

Timeframe	Number of Wounding-Related Events, Severity ^1^	Temperaments of Individuals Involved in Incidents	Remarks
Initial grouping	2, minor	Fearful, Aggressive	Two interconnected pens used
Month 1	11, minor 2, moderate	All temperaments	
Month 2	3, minor	Fearful, Non-aggressive	
Month 3	4, minor	Aggressive, Non-aggressive	
Month 4	1, minor	Aggressive	
Month 5	1, minor	Aggressive	
Month 6	2, minor	Aggressive	B, F removed for study; one pen used
Month 7	4, minor 1, severe	Aggressive, Non-aggressive	
Month 8	None		
Month 9	None		Two interconnected pens used
Months 10–12	1, minor 1, moderate	Aggressive, Non-aggressive	
Month 13–15	4, minor 2, moderate 1, severe	Aggressive, Non-aggressive	H removed permanently
Months 16–18	5, minor	All temperaments	
Months 19–25	None		
Months 26–30	7, minor 2, moderate	All temperaments	

^1^ Wounds that were categorized as moderate or severe required clinical intervention.

**Table 3:** Temperament of each aged *Macaca fascicularis* subject based on PAIR-T formal assessment, clinical diagnosis and body-weight condition.

Subject ID	Temperament	Clinical Diagnosis	Pre-Social Housing Body Weight (kg)	Post-Social Housing Body Weight (kg)
1	Aggressive	Non-diabetic	7.18	6.49
2	Neutral	Pre-diabetic	6.40	5.70
3	Neutral	Pre-diabetic	5.21	4.06
4	Neutral	Pre-diabetic	7.24	6.19
5	Affiliative	Non-diabetic	6.46	5.69
**6**	Anxious	Non-diabetic	5.42	5.79

**Table 4:** Clinical incidents documented in the aged cohort of male *Macaca fascicularis* during social housing attempts.

Type of Social Housing Attempt (Subjects ID)	Approximate Duration	Number of Wounding-Related Events, Severity ^1^	Injured Subject ID	Remarks
Group	1 month	2, minor 2, moderate 2, severe	1, 3, 4, 5, 6	Subjects 1,4 removed for treatment
Pair (ID: 1-5; 2-6; 3-4)	2 months	1, minor 3, moderate	1, 2, 4, 5	
Trio (ID: 2-5-6; 1-3-4)	1 month	2, minor 1, moderate	1, 4, 6	
Group	1 week	2, minor 2, moderate	1, 2, 4, 5	
Trio (ID: 2-5-6; 1-3-4)	3 months	1, minor 1, moderate 1, severe	1, 4	Subject 1 removed for treatment
Group	1 month	3, minor 1, moderate 1, severe	1, 2, 4	
Trio (ID: 2-4-6; 1-3-5)	7 months	1, minor	4	

^1^ Wounds of moderate and severe categories required clinical intervention.

**Table 5:** Dominance and temperament identification of each aged *Macaca fascicularis* cohort subject when socially housed, based on cage-side observation and/or hand-feeding reactions.

Subject ID	Dominance	Temperament
1	Subordinate, higher rank	Aggressive
2	Subordinate, higher rank	Neutral
3	Dominant	Neutral
4	Dominant	Neutral
5	Subordinate, lower rank	Affiliative
6	Subordinate, lower rank	Anxious

## Data Availability

Data supporting the reported results are available within the article and upon request to the corresponding author.
